# Nutritional profiling and sensory evaluation of hilsa *Tenualosa Ilisha* (Hamilton, 1822) eggs roe from three different locations of Bangladesh

**DOI:** 10.1038/s41598-025-16332-7

**Published:** 2025-08-22

**Authors:** Muhammad Anamul Kabir, Kamrul Islam, Shishir Kumar Nandi, Sanzida Hoque, El-Sayed Hemdan Eissa, Hien Van Doan, Suniza Anis Mohamad Sukri, Zulhisyam Abdul Kari

**Affiliations:** 1 Department of Aquaculture, Faculty of Fisheries, Sylhet Agricultural University, Sylhet, 3100 Bangladesh; 2 Advanced Livestock and Aquaculture Research Group, Faculty of Agro- Based Industry, Universiti Malaysia Kelantan, Jeli Campus, 17600 Jeli, Malaysia; 3 Fish Research Centre, Faculty of Environmental Agricultural Sciences, Arish University, El-Arish, 45516 Egypt; 4 Department of Animal and Aquatic Sciences, Faculty of Agriculture, Chiang Mai University, Chiang Mai, 50200 Thailand; 5 Functional Feed Innovation Centre (FuncFeed), Faculty of Agriculture, Chiang Mai University, Chiang Mai, 50200 Thailand; 6 Department of Agricultural Sciences, Faculty of Agro-Based Industry, Universiti Malaysia Kelantan, Jeli Campus, 17600 Jeli, Malaysia

**Keywords:** Food security, Amino acids, Fatty acids, Minerals, Sensory test, Hilsa eggs roe, Biochemistry, Biotechnology

## Abstract

Hilsa (*Tenualosa ilisha*) eggs roe is considered delicacy food products among consumers and has high potential due to its superior nutrients. This study, conducted in 2023, examined the nutritional profiling and sensory attributes of *T. ilisha* eggs roe from Cox’s Bazar, Chandpur, and Patuakhali regions in Bangladesh. Majority of the bioimetrical and morphometric parameters were significantly (*P* < 0.05) varied across distinct areas. The eggs roe proximate composition were significantly (*P* < 0.05) influenced by different areas. Significant (*P* < 0.05) variations were found in most of the amino acid contents in Hilsa eggs from distinct locations. Glutamic acid (14.20–14.60%) was the predominant amino acid in all locations, followed by alanine (12.77–13.03%), aspartic acid (8.60–9.50%), and leucine (8.43-9.00%). Among 22 identified fatty acids in Hilsa eggs roe, C16:0 (31.08–32.86%), C18:1n-9 (20.73–22.23%), and C22:6n-3(5.55–5.68%) dominated as major SFA, MUFA, and PUFA, respectively. Significantly (*P* < 0.05) the highest Σω-3 were detected in the egg’s roe from Patuakhali. The mineral composition showed significant (*P* < 0.05) differences across diverse locations. The eggs roe from Patuakhali had significantly (*P* < 0.05) increased taste and appearance. Hence, these findings suggested that eggs roe development from Patuakhali region could be better compared to other area for human health benefit in terms of nutritional point of view.

## Introduction

The Hilsa fish (*T. ilisha)*, an anadromous species that undertakes a remarkable migration from marine environment to rivers for the purpose of spawning, is a vital resource for fisheries in several South-Asian countries, especially in Bangladesh^1^. This species is found across a broad habitat, ranging from the Persian Gulf to the Bay of Bengal^2^. The Hilsa, belonging to the Clupeidae family, is commonly known as “Ilish” in Bangladesh^3^. This renowned fish species is regarded as an iconic and economically significant fish, appreciated for its delectable taste, flavor, and impressive nutritional profile^4^. The national fish of the country, Hilsa, was officially recognized and registered as a Geographical Indication (GI) product in 2017 by the government of Bangladesh^5^. Reports indicate that Hilsa, as a single species, contributed to around 12% of the total fish production^6^ with an impressive 65% of the global Hilsa catch originating from Bangladesh’s marine environment^7^ showing the country’s commitment for a stable food security strategy^8^.

Hilsa is a fatty oily fish, brimming with a plethora of essential nutrients. It is plentiful with essential fatty acids & amino acids that promote brain development and nervous system, lower cholesterol level, and support tissue repair for public health benefit^9^. In general, nutritional composition of the fish varies depending on the factors like species, habitat, feeding behavior, and the availability of food^10–14^. Furthermore, the flavor and taste of fish are remarkably influenced by their living conditions, dietary preferences, and the biochemical makeup of their bodies^9^. Among the edible parts of fish, roe (fish eggs) is a particularly valuable tissue with distinctive biochemical properties compared to muscle or other organs. The term “roe” is referred to the eggs of female broods and can originate from species of fish including tuna, herring, cod, trout, whitefish, salmon, or even carp and shellfish^4,15–18^. Fish eggs exhibit variations in size, color, and texture. Roes are globally cherished food item and primarily prepared through methods like cooking, frying, or grilling^11,17^. Notably, eggs of Hilsa hold immense popularity as a distinct culinary food product during various festivals in Bangladesh and esteemed as a delicacy in many parts of the world. Beyond cultural significance, fish roe is gaining scientific attention due to its exceptionally high contents of essential macronutrients and micronutrients that are crucial for human nutrition and health. However, scientific data specifically focusing on Hilsa roe remain scarce, underscoring the need for detailed nutritional profiling of this tissue.

The fish eggs primarily consist of protein and lipid, with over 50% crude protein and 30% crude lipid on a dry matter basis and serve as valuable reservoirs of essential amino acids, fatty acids, and minerals^9,19–21^. Fish eggs are abundant in dietary source of essential amino acids like leucine, lysine, valine, and arginine, vital for body building, tissue repair, reproductive development, and energy provision in human health^11,22^. Meanwhile, the major constituent of fish eggs lipid is n-3 PUFA such as docosahexaenoic acid (DHA) and eicosapentaenoic acid (EPA), offers health benefits like supporting brain and visual development while mitigating the risk of coronary heart disorders^9,23,24^.

Several researchers^3,9–11,25^ have extensively studied the nutritional profile of Hilsa shad muscle, primarily focusing on larger fish groups and their potential benefits for human health. Additionally, multitude of prior studies have developed eggs roe or caviar from several fish species including Bigeye tuna^13^ Cod^14^ Pollock^26,27^ Sturgeon^28–33^ and Salmon^34,35^. Surprisingly, there is a notable gap in research when it comes to the development of roe or caviar from the Hilsa fish. Furthermore, most studies have overlooked how environmental factors such as habitat (marine, brackish, or freshwater) may influence the nutritional composition of Hilsa roe. A significant deficiency exists in systematically exploring the nutritional contents of Hilsa fish eggs across diverse locations in the Southern part of Bangladesh. Therefore, understanding the critical nutrient requirements for the development of Hilsa female brood eggs is essential for preparing high-quality roe that not meets consumer expectations, but can provide additional nutrition for better human health. This study is novel in addressing both the nutritional and sensory aspects of Hilsa roe with respect to habitat-specific variation, an area that has not been previously investigated.

This uncharted territory in roe or caviar production from Hilsa fish holds great potential for scientific inquiry, commercial ventures and food security. Hence, this current study aimed to assess the nutritional profile of female Hilsa brood eggs sourced from different locations, including Cox’s Bazar (marine), Patuakhali (brackishwater), and Chandpur (freshwater), with a focus on assessing variations in the nutritional content of Hilsa eggs, including protein, lipid, ash, amino acids, fatty acids, and mineral contents. In addition, the study evaluated sensory properties, providing a complete profile that links nutrient richness with consumer acceptability. The data produced would be very valuable in clinical nutrition for aquaculture nutritionists and dieticians, serving as a reference to recommend the use of Hilsa fish eggs from specific locations for specialized roe preparation, commercialization, public health promotion and food security.

## Materials and methods

### Sampling site

**Fig. 1:**
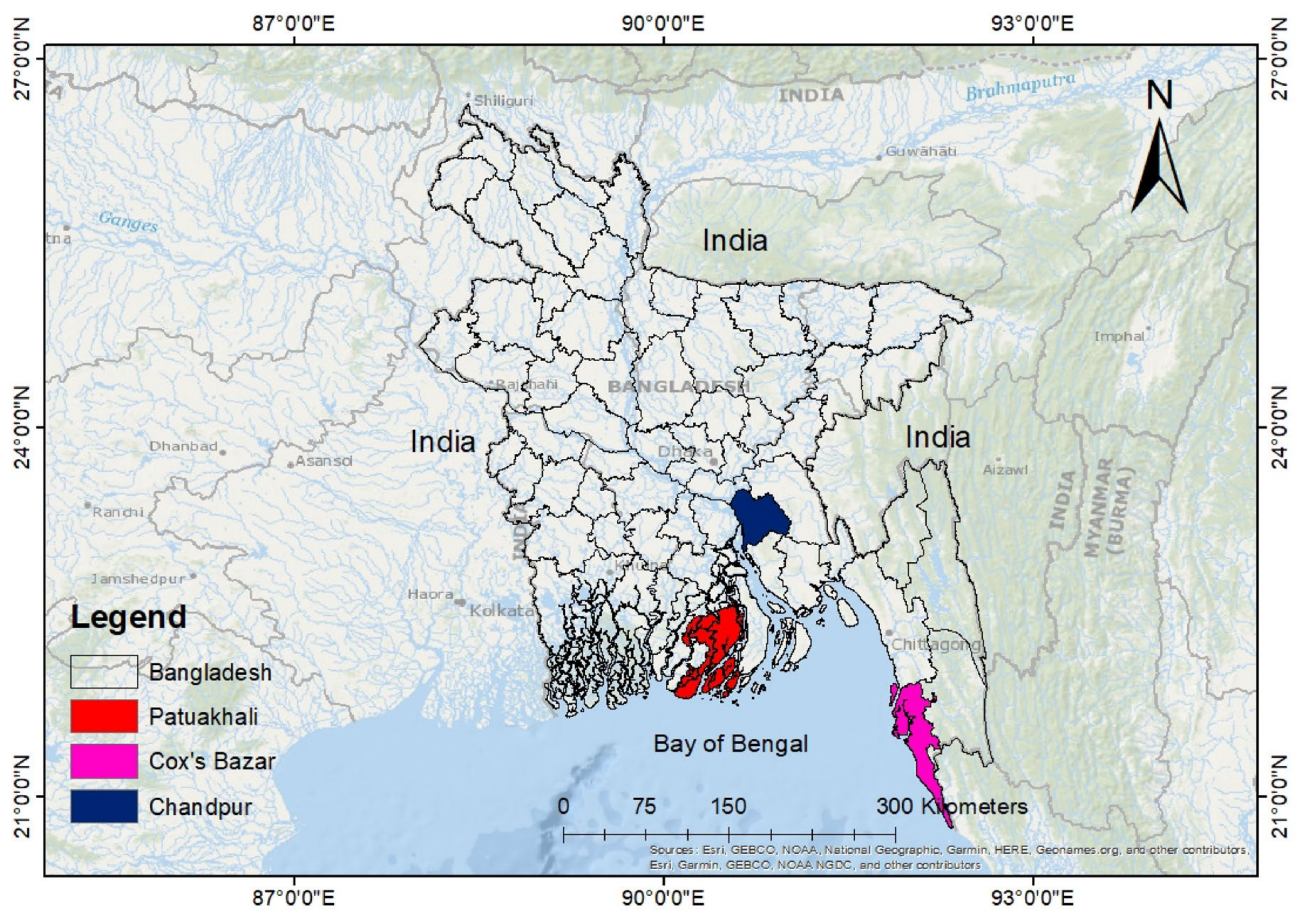
Sampling sites of *T. ilisha* in Bangladesh, showing the three major collection locations: Cox’s Bazar (marine), Patuakhali (brackish water), and Chandpur (freshwater).

This research was directed in marine, estuary and riverine areas of Cox’s Bazar, Patuakhali, and Chandpur respectively (Fig. 1), which are the three major regions for capturing *Tenualosa* sp. in Bangladesh. Cox’s Bazar (21°24′−21° 36′ N and 91° 59′–92° 08′ E) is situated along the Bay of Bengal coastline. Chandpur (20° 43′−20° 43′ N and 90° 35′–90° 39′ E) lies between the Padma and Meghna River systems, serving as breeding, feeding and nursing ground for Hilsa fish. Patuakhali (22° 22′−22° 35′ N and 90° 20′–90° 33′ E) is situated along the bank of Laukathi and Lohalia, two major river systems. Samples were collected from fish landing centers (fish arats) in each region: BFDC Ghat in Cox’s Bazar, Boro Station in Chandpur, and Mohipur Fish Landing Center in Patuakhali. Fish were obtained immediately after landing at these centers rather than directly from fishing boats. Although marine and brackish water Hilsa may sometimes be landed at the same centers, especially in Patuakhali, fishers and local agents (aratdars) were consulted to ensure the origin of the specimens. Samples from Patuakhali were confirmed to be from brackish water systems based on fishing reports.

### Fish collection and sampling of eggs roe

A total of 90 Hilsa *T. ilisha* (30 fish from each location) was collected from three fish landing center in the South-East part of Bangladesh: Cox’s Bazar, Chandpur and Patuakhali in January to February 2023. Upon arrival at each different location, fresh Hilsa fish were collected and placed immediately in insulated containers containing crushed flake ice, maintaining a 4:1 ratio of fish to ice for preservation. Subsequently, the Hilsa fish were conveyed to the Laboratory in order to record their individual body weight and length, facilitating the measurement of various biometrical parameters of each fish such as body weight (BW), total length (TL), body depth (BD), liver weight (LW), hepatosomatic index (HSI), viscera weight (VW), and visceral somatic index (VSI). The fish were dissected ventrally using scissors from anus to mouth. Following that, the eggs roe was carefully extracted from each fish and the dimension and weight of each separated egg roe were taken to measure their morphometric parameters including right side total length of egg roe (RSTL), right side anterior depth of egg roe (RSAD), right side middle depth of egg roe (RSMD), right side posterior depth of egg roe (RSPD), left side total length of egg roe (LSTL), left side anterior depth of egg roe (LSAD), left side middle depth of egg roe (LSMD), left side posterior depth of egg roe (LSPD). Reproductive indices such as ovarian weight and Gonadosomatic index (GSI) for each located fish in this study were computed as per Nandi et al.^36^ and the following formulae were used:

Gonadosomatic index, GSI = 100 × (Gonad weight / Fish body weight).

Visceral somatic index, VSI = 100 × (Viscera weight / Fish body weight).

Hepatosomatic index, HSI = 100 × (Liver weight / Fish body weight).

The sampled eggs were preserved at − 20 °C, dried to constant weight using a hot air oven (Model: BOV-V30F, BIOBASE, China), finely ground using a grinder (Model: MX-AC400, Panasonic, Japan), and stored again at − 20 °C in airtight containers until further analysis was conducted following standard analytical procedures.

### Biochemical composition analyses of eggs roe

The analysis of Hilsa eggs biochemical composition followed the established procedures outlined by the AOAC^37^ with few alterations. Crude protein was determined using the Kjeldahl method with a Kjeldahl digestion unit. About 0.5 g of each finely ground, dried egg sample was weighed into digestion tubes. A catalyst mixture consisting of potassium sulfate and copper sulfate in a 9:1 ratio was added, followed by 25 ml of concentrated sulfuric acid (H₂SO₄). The samples were then digested at a temperature range of 350–380 °C until a clear solution was obtained, indicating the conversion of organic nitrogen into ammonium sulfate. After cooling, the digested samples were diluted with approximately 300 ml of distilled water. During distillation, 100 ml of 35% sodium hydroxide (NaOH) was added to liberate ammonia, which was then captured in a 25 ml boric acid solution (4%) containing a mixed indicator (bromocresol green and methyl red) placed in a receiving flask. The distillation was continued until about 75 ml of distillate was collected. Finally, the trapped ammonia was quantified through titration with 0.1 N hydrochloric acid (HCl) until the color of the solution changed from green to pink. The amount of nitrogen was calculated from the titration values, and the crude protein content was expressed as a percentage using the standard conversion factor (%*N* × 6.25).

Crude lipid was determined using a Soxhlet apparatus. Approximately 3 g of dried, ground sample was placed in a filter thimble and inserted into the Soxhlet chamber. Around 150–180 ml of acetone was added to the round-bottom flask and the system was heated at 70 °C for 3 h to extract lipids through continuous solvent cycling. The lipid-containing solvent was then transferred to a pre-weighed beaker and dried in an oven at 105 °C for 30 min to evaporate acetone. After cooling in a desiccator, the beaker was re-weighed. Lipid content (%) was calculated based on the weight difference.

Ash content was determined using a muffle furnace. Pre-weighed empty crucibles were labeled, and around 2 g of each dried sample were added and weighed. The crucibles were then heated in the furnace at 550 °C for 6 h to combust organic matter. After cooling overnight, samples were further cooled in a desiccator before being weighed again. Ash percentage was calculated from the weight difference.

Moisture content was measured using a hot air oven. Pre-weighed crucibles were labeled, and about 2 g of each sample were added. The crucibles were dried in the oven at 105 °C for 24 h. After drying, they were cooled in a desiccator for 15 min before being weighed again. Moisture percentage was calculated based on the weight loss.

### Amino acid analyses of eggs roe

The analyses of amino acids profile in Hilsa eggs roe was conducted following a recognized method as delineated by AOAC^37^ and Abdul Kari et al.^38^ with some modifications applied. Shortly, the amino acid contents of each test sample were ascertained after being subjected them to hydrolysis using 6 N HCL at 110 °C for 24 h. Subsequently, the derivatization process was performed using AccQ Fluor reagent (6-aminoquinolyl-N-hydroxysuccin-imdylcarbamate) and chromatographic separation was accomplished on an AccQTagTM reversed phase analytical column with dimensions of 3.9 × 150 mm (length x inner diameter). To conduct amino acid profiling, a high-performance liquid chromatography (HPLC) (Model: Waters 2695, Waters Corporation, USA), was utilized, which included water 1525 binary HPLC pump, a 717 plus auto-sampler and waters 2475 Multi λ Fluorescence detector set to wavelengths of 250 nm for detection and 395 nm for emission. Alpha-amino butyric acid (AABA) was employed as an international standard for measuring amino acids in sample. Furthermore, Elution was facilitated by Acetonitrile and AccQTagTM. The chromatographic peaks were integrated, identified, and quantified through the use of BreezeTM software version 3.20, which was compared against established standards (amino acid standard H, Pierce Rockford, Illinois, USA) for matching and verification. The test samples were analyzed in triplicate for each analysis. The amino acid was calculated using the following formula:

Amino acids (%) = 100 × (Amount of amino acids/ Sample weight).

### Fatty acid analyses eggs roe

The fatty acids profile of Hilsa eggs were determined following the methodology outlined by Kabir et al.^20^. For this purpose, the eggs roe sample were first frozen at -20 °C and subsequently underwent to drying. The dried samples were finely pulverized, individually placed in screw-capped bottle, and preserved at -20 °C fewer than 3 days before being analyzed for their fatty acid contents. The experiment was carried out in triplicates. The synthesis of fatty acid methyl esters (FAMEs) was achieved through a one-step extraction transesterification method. In short, about 100 mg egg sample from each were measured into 10 ml screw top glass container and combined with a solution of 2 ml methanol and sulfuric acid (V: V, 85:15), along with 2 ml chloroform. Each bottle was subjected to vortexing for 30 s using a vortex mixture to infuse nitrogen gas and then sealed with Teflon caps to prevent leakages. Each replicate sample was exposed to a temperature of 100 °C for 30 min and subsequently allowed to cool down to room temperature (25 °C). Afterward, 1 ml of distilled water was mixed with the solution and vigorously vortexed for 1 min. Following the emergence of two distinct phases, the lower phase holding FAMEs was extracted and subjected to dehydration using anhydrous sodium sulfate. The samples were subjected to freezing at -20 °C until they were ready for gas chromatographic analysis. To prepare the sample for Gas Chromatography (GC) injection, 1 µl of internal standard caproic acid methyl ester, which was diluted in chloroform at a ratio of 1:499 (V: V) with 3 µl dried sample in a vial. Subsequently, about 1 µl of the resulting sample was injected in the GC. Gas chromatography (Automatic System XL by Perkin Elmer, USA) equipped with a flame ionization detector and a 30 m x 0.25 mm fused silica capillary column (Megawax 250, Supelco, USA) was utilized to separate and quantify FAMEs. Helium served as carrier gas, while hydrogen along with compressed air was employed for flame ionization detection (FID). The temperature in the oven was programmed to ascend by 4 °C per minute, moving from 50 to 220 °C and then maintained at 220 °C for 35 min. The injector temperature was set to 250 °C, while the detector temperature was adjusted to 260 °C. Then, the chromatographic data were captured and processed on a computer employing Turbochrom software (Perkin Elmer, USA). All fatty acids were distinguished by comparing the retention time of its FAMEs, with those found in standard component FAMEs mixture and menhaden oil. The amount of each fatty acid was quantified as a proportion of the total detected fatty acids in the sample. Fatty acid was determined employing the following formula:

Fatty acid (%) = 100 × (Peak area of each fatty acid/ Total peak area of all fatty acids).

### Mineral analyses eggs roe

The analysis of mineral components of Hilsa eggs roe was carried out according to the standard protocol elucidated by AOAC^37^ with slight alterations. In brief, about 5 g of the eggs sample from each group was meticulously weighed into an acid-washed crucible and subjected to over-drying at 105 °C for 24 h. Subsequently, the dried eggs roe samples underwent to incineration within a Muffle furnace at 550 °C overnight. The resulting ash content was subsequently digested with 5 ml of 65% nitric acid, boiled for two min, and then allowed to cool to room temperature. Following that, the solution was filtered through Whitman filter paper (NO: 41), with subsequent dilution to 25 ml 65% nitric acid. From this prepared solution, 10 ml was transferred into a polypropylene test tube for injection into an inductively-coupled plasma-optical emission spectrometer (ICP-OES) (Perkin Elmer, USA). The micro-minerals including potassium (K) (wavelength: 766.491, view type: radial), phosphorus (P) (wavelength: 178.29, view type: axial), magnesium (Mg) (wavelength: 383.83, view type: axial), calcium (Ca) (wavelength: 317.93, view type: radial), manganese (Mn) (wavelength: 257.61, view type: axial), iron (Fe) (wavelength: 259.94, view type: axial), copper (Cu) (wavelength: 324.75, view type: axial), and zinc (Zn) (wavelength: 213.86, view type: axial), were determined. A sample black containing 65% nitric acid was analyzed simultaneously with each batch of samples. The results obtained were quantified by comparing them against a multi-element standard (Perkin Elmer, AVIO 200, Waltham, USA).

### Sensory evaluation test of cooked hilsa eggs roe

Sensory evaluation was carried out on Hilsa eggs roe, employing a nine-point hedonic scale to compare various attributes across distinct geographical sources of Hilsa. A panel of 30 evaluators, consisting of 10 undergraduate students (age range: 20–23), 15 postgraduate students (age range: 24–27), and 5 individuals holding PhD degrees (age range: 45–50), was chosen for the assessment. The evaluators were all in good health with no reported allergies or sensitivities to fish or seafood products. Prior to the evaluation, the panelists underwent a brief screening and orientation session to familiarize them with the sensory attributes and scoring method to ensure consistency and reliability of responses. The Hilsa roe samples were prepared using a traditional pan-frying method, commonly practiced in Bangladeshi cuisine. For each sample group, approximately 100 g of fresh roe was mixed with 1.0 g of freshly ground ginger paste, a pinch of salt (about 1.0 g), and no additional spices or condiments to minimize external flavor interference during sensory evaluation. The samples were then fried in 10 ml of refined soybean oil using a non-stick frying pan. Cooking was conducted at a controlled temperature of 170–180°C for 5–6 minutes, turning the samples occasionally to ensure even browning and complete cooking. After frying, the samples were removed from heat, allowed to cool down for 2–3 minutes, and brought to room temperature (25°C). Excess oil was removed by placing the samples on absorbent paper. The fried samples were then presented to each evaluator for sensory assessment. A structured questionnaire was designed, utilizing a nine-point hedonic scale ranging from ‘like extremely’ to ‘dislike extremely’^11^. This scale allowed panelists to rate their preferences for six key parameters: Appearance, Taste, Texture, Sweetness, Aroma, and Overall Acceptability. Each panelist evaluated all samples to ensure comprehensive and comparative analysis of the roe products. Each sensory evaluation was performed in triplicate to ensure repeatability and data consistency.

### Statistical analysis

The biometric, reproductive, proximate, amino acids, fatty acids, mineral contents, and sensory assessment of Hilsa eggs were analyzed using SPSS software version 27.0. A one-way analysis of variance (ANOVA) was conducted to ascertain the mean values of individual parameter and the statistical variations (*P* < 0.05) were identified using Duncan’s multiple range test. Mean values with distinct superscripts specify remarkable variations.

## Results and discussion

### Bimetrical, reproductive and morphometric parameters

The biometrical, reproductive, and morphometric indices of Hilsa (*T. ilisha*) sourced from three different locations of Bangladesh are summarized in Table 1. The findings illustrated significant (*P* < 0.05) variability in biometrical parameters such as BW (g), TL (cm), BD (cm), LW (g), HSI, VW (g), and VSI among the fish from different localities. In particular, increased levels of these parameters were observed in fish from Cox’s bazar area. These results might be attributed to the dissilimar size of fish that are captured during this research. When *Siganus rivulatus* sampled from different locations in the Egypt showed significant variations of their TL and BW^36^. *O mykiss* collected from various stations in Turkey revealed similar variations of BW and TL^37^. Idential variation of these parameters were also noted in *Tenualosa* sp^37^. and Sperata sp^39^. In contrast, Fazio et al.^39^. Hossain et al.^3^ and Kabir et al.^21^ reported no substantial variations of these variables when *Gobius niger* captured from Faro lake aand Tyrrhenian Sea, *T. ilisha* from Bay of Bengal and Arabian Gulf, and *S. aor* from wild and captive sources of Bangladesh respectively. However, the SL (cm) of Hilsa fish across varous locations didn’t vary significantly (*P* > 0.05) in this research.

The GW and GSI are valuable indices that are used to assess the reproductive status and maturity of fish^20^. In fish reproductive biology, GSI represents the proportion of a fish’s GW to its total BW. In this study, geographical areas had significant (*P* < 0.05) impacts on both GW (71.70 ± 3.80–93.60 ± 5.40 g) and GSI (7.05 ± 0.54–10.83 ± 1.09), with markedly elevated values were observed in the fish group belonging from the Patuakhali region when compared to those from other regions. Patuakhali, being a brackish water zone, provides an optimal balance of salinity and temperature which may stimulate gonadal development. Similar variations of these parameters were also noted in other fishes such as *Seriola dumerili*^40^ *Sperata aor*^21^ *Mugil cephalus*^41^. The value of GSI, subject to seasonal variation, offers valuable insights into a fish’s reproductive cycle, aiding discern peak spawning period and reproductive pattern.

Additionally, the eggs morphometric data, including RSTL (13.80 ± 0.29 cm), RSPD (2.18 ± 0.07 cm), LSTL (13.84 ± 0.27 cm), LSAD (2.00 ± 0.07 cm), LSMD (3.26 ± 0.11 cm), and LSPD (1.77 ± 0.18 cm), exhibited significantly (*P* < 0.05) greater values in the Hilsa fish sampled from the Patuakhali region in comparison with other areas. Nonetheless, the experimental fish from various locations had no significant (*P* > 0.05) impact on other metrices like RSAD and RSMD. The observation of these parameters provides adequate information about gonadal structure and reproductive status of Hilsa fish. These location-based variations imply environmental influences on eggs characteristics. This understanding informs conservation and habitat management, vital for Hilsa’s sustainable management and protection while enhancing the grasp of its reproductive ecology.

**Table 1:** Bimetrical, repdorductive, and morphometric variables of experimental fish from three geographical areas of Bangladesh (*n* = 30).

Parameters	Locations		
	Cox’s Bazar	Chandpur	Patuakhali
Biometrical parameters			
BW (g)	1030.37 ± 4.41^a^	930.33 ± 10.79^b^	863.87 ± 31.79^c^
TL (cm)	41.70 ± 0.10^a^	41.10 ± 0.30^b^	40.97 ± 0.31^b^
SL (cm)	34.47 ± 0.06	34.37 ± 0.15	34.17 ± 0.21
BD (cm)	12.12 ± 0.22^a^	11.03 ± 0.12^b^	10.63 ± 0.01^c^
LW (g)	28.10 ± 0.53^a^	8.73 ± 0.25^b^	9.20 ± 0.35^b^
HSI	2.74 ± 0.04^a^	0.94 ± 0.02^c^	1.01 ± 0.06^b^
VW (g)	49.03 ± 1.56^a^	24.07 ± 0.45^b^	26.00 ± 0.76^b^
VSI	4.77 ± 0.17^a^	2.59 ± 0.09^c^	3.04 ± 0.06^b^
Repdorductive parameters			
GW (g)	72.93 ± 5.66^b^	71.70 ± 3.80^b^	93.60 ± 5.40^a^
GSI	7.05 ± 0.54^b^	7.67 ± 0.35^b^	10.83 ± 1.09^a^
Eggs roe morphometric parameters			
RSTL (cm)	12.58 ± 0.07^b^	14.11 ± 0.23^a^	13.80 ± 0.29^a^
RSAD (cm)	1.72 ± 0.07	1.71 ± 0.02	1.77 ± 0.08
RSMD (cm)	2.65 ± 0.02	2.69 ± 0.13	2.89 ± 0.31
RSPD (cm)	1.61 ± 0.05^c^	1.84 ± 0.05^b^	2.18 ± 0.07^a^
LSTL (cm)	11.82 ± 0.06^b^	13.64 ± 0.06^a^	13.84 ± 0.27^a^
LSAD (cm)	1.60 ± 0.07^b^	1.67 ± 0.05^b^	2.00 ± 0.07^a^
LSMD (cm)	2.76 ± 0.05^b^	2.82 ± 0.02^b^	3.26 ± 0.11^a^
LSPD (cm)	1.63 ± 0.06^b^	1.67 ± 0.02^b^	1.77 ± 0.18^a^

The data are represented as mean ± sd of means.

BW: Body weight, TL: Total length, SL: Standard length, BD: Body depth, LW: Liver weight, HSI: Hepatosomatic index, VW: Visceral weight, VSI: Visceral-somatic index, GW: Gonad weight, GSI: Gonadosomatic index, RSTL: Right side total length of egg roe, RSAD: Right side anterior depth of egg roe, RSMD: Right side middle depth of egg roe, RSPD: Right side posterior depth of egg roe, LSTL: Left side total length of egg roe, LSAD: Left side anterior depth of egg roe, LSMD: Left side middle depth of egg roe, LSPD: Left side posterior depth of egg roe. Mean values with distinct superscripts specify remarkable variations.

### Biochemical composition of hilsa eggs roe

Table 2 illustrates the protein, lipid, ash and moisture contents of Hilsa eggs. Results indicated that nutritional profile of Hilsa eggs were significantly (*P* < 0.05) influenced when obtained from different locations during this study. Several factors including fish type, age, season, geographical area, and habitat have critical role in influencing the nutritional composition of fish eggs, as reported by Debnath et al.^42^, Begum et al.^43^, and Kowalska-Góralska et al.^44^. Moreover, the nutritional contents of female ovary serves as a measure of eggs quality^21,45^. Moisture, as the non-nutrient component of fish eggs, serves as a valuable indicator of the eggs’ nutritional value. To be more specific, increased moisture content correponds to reduced nutritional worth. Based on the current findings, it was found that that the percentages of moisture (4.51 ± 0.49%) in Hilsa eggs from Cox’s Bazar was significantly (*P* < 0.05) lower, indicating greater nutrient levels in comparison with eggs from other areas. Comparable fluctuations in moisture contents (61.12–67.26%) were observed in the *T. ilisha* eggs across 4 distinct regions in Bangladesh^46^.

As a fundamental macronutrient, protein content reflects the nutritional richness and consumer acceptability of food products. The protein quantity in eggs exhibited significant variability across the different locations. The significantly higher protein content (65.57 ± 0.15%) observed in Hilsa eggs from Cox’s Bazar may be due to the pre-spawning physiological stage of marine Hilsa, supported by a protein-rich diet, active vitellogenin synthesis, and the absence of migratory stress. These combined factors create favorable conditions for enhanced protein syntheis in the developing oocytes. Analogous protein content was also identified within the eggs of several fish species such as Salmon *Salmo truttalabrax* and Mullet *Mugil cephalus* (24.0–27.3%)^47^ Rainbow trout *Oncorhynchus mykiss* (20.60–25.43%)^48^ Siberian sturgeon *Acipenser baeri*, Rainbow trout *Oncorhynchus mykiss*, and Siberian sturgeon *Acipenser baeri*, Rainbow trout *Oncorhynchus mykiss*, and Sturgeon hybrid *Acipenser baeri x Acipenser gueldenstaedti* (19.82–24.93%)^44^ and Long whiskered *Sperata aor* (50.33–51.31%)^21^.

Lipid acts as a stored energy for fish^49,50^ supporting gonadal development and are utilized during long distance migration. The lipid level of *T. ilisha* eggs exhibited significance among the fish groups (*P* < 0.05), ranging from 24.46 to 25.38% in this study. The Rainbow trout eggs displayed similar lipid variations (3.19–5.76%) while collecting from various stations in Turkey^48^. Likewise, other research works also supported this discovery^34,44,48,51,52^. Furthermore, fish eggs from Chandpur and Patuakhali regions contained remarkably (*P* < 0.05) lower lipid, might be attributed to energy depletion associated with the upstream migration of Hilsa.

In addition, the Hilsa oocytes ash content (4.61 ± 0.05%) was significantly (*P* < 0.05) higher in Patuakhali fish as compared to other sampling sites, which is identical with the earlier studies in the eggs of other fishes^44,46,48,53^. This result might be credited to different availability of minerals in diverse environment. Kabir et al.^45^ pointed out that an increased concentration of inorganic phosphate plays a crucial role as an ion necessary for the synthesis of adenosine tri-phosphate (ATP), nucleic acid, and glycolysis in eggs of fish. Overall, the observed variations in the composition of Hilsa eggs at various locations could be due to variations in habitat, geographical area, water environment, and food availaibilty.

**Table 2:** Biochemical composition (% dry matter) of hilsa Shad eggs belonging to three distinct locations of Bangladesh (*n* = 3).

Parameters	Locations		
	Cox’s Bazar	Chandpur	Patuakhali
Moisture	4.51 ± 0.49^b^	5.56 ± 0.52^a^	5.66 ± 0.51^a^
Protein	65.57 ± 0.15^a^	63.87 ± 0.45^b^	63.16 ± 0.51^c^
Lipid	25.38 ± 0.24^a^	24.60 ± 0.04^b^	24.46 ± 0.11^b^
Ash	4.06 ± 0.14^b^	4.15 ± 0.02^b^	4.61 ± 0.05^a^

he data are represented as mean ± sd of means.

Mean values with distinct superscripts specify remarkable variations (*P* < 0.05).

### Amino acid profile in hilsa eggs roe

Table 3 mentions the amino acid composition of eggs of *T. ilisha* collected from different geographical areas of Bangladesh. The primary function of amino acids (AAs) is to act as a building block of protein^54^. Fishes require both essential and non-essential amino acids to support their somatic growth, maturation, and reproductive activity and these AAs are used in various biological processes, including protein and peptide synthesis, ATP synthesis, biological oxidation, anti-oxidative reactions, and immune functions^54^.

The present outcomes indicated that NEAAs such as aspartic acid, serine, glutamic acid, glycine, and cysteine contents showed significant (*P* < 0.05) alterations across the various located Hilsa fish eggs, except for alanine content. The observed differences in NEAAs likely result from environmental variability (marine, brackish, freshwater), dietary influences, and differing reproductive developmental stages affecting fish metabolic activity and protein biosynthesis pathways. Similar observations were reported in the eggs of *Hilsa ilisha*^49^ and *Oncorhynchus mykiss*^48^. Among the NEAAs, glutamic acid (14.20–14.60%) was found to be the most prevalent across all locations, trailed by alanine (12.77–13.03%), and aspartic acid (8.43-9.00%). According to Baki et al.^48^ Rainbow trout eggs collected from various stations in Turkey were found to contain approximately 3.05–4.54% glutamic acid (Glu), 2.26–3.27% alanine (Ala), and 2.37–3.47% aspartic acid (Asp). Additionally, in *S. aor* from both wild and farmed sources, their eggs contained varying levels of Glu (5.50-10.49%), Ala (5.72%), and Asp (2.78–2.79%)^21^. Conversely, Cysteine (Cys) constituted the least prevalent NEAAs at a range of 1.60–2.80%. This finding is similar with Pal et al.^49^ discovery of *H. ilisha* eggs having approximately 0.59% Cys.

Fish eggs are regarded as highly nutritious due to their balanced protein and EAA contents^55^. Fish eggs have distinct amino acids profile compared to fish muscle^56,57^. In this observation, the sampled female Hilsa from various locations had significant (*P* < 0.05) variations in the levels of several essential amino acids (EAAs), particularly threonine, valine, methionine, isoleucine, leucine, tyrosine, and histidine. However, these variations did not follow any specific or consistent trend. Amongst all the EAAs, leucine (8.43-9.00%), lysine (7.30–7.60%), threonine (4.90–6.20%), and arginine (5.80–6.20%) were the most prominently represented amino acids in the eggs of each regional fish group. These EAAs are vital for human nutrition and their presence in Hilsa fish eggs could suggest potential dietary benefits for human consumption and improve nutrition availability. The predominance of these amino acids in Hilsa eggs across all regions, reflecting their crucial roles in yolk protein synthesis, embryonic development, immune defense, and enzymatic activities. Previous reports have shown that leucine (Leu) was the most dominated AAs in different fish eggs^48,58,59^ which are strongly correlated with this finding.

Additionally, the mean values of ΣNEAA, ΣEAA, EAA/NEAA were statistically varied (*P* < 0.05) across the various Hilsa groups, with ranges of 49.17–50.53%, 49.47–50.83%, and 0.98–1.03 respectively (Table 3), indicating that different geographical locations had substantial effects on these variables. Baki et al.^48^ also found significant variations in these parameters. However, in this study, other EAAs like phenyl-alanine, lysine, and arginine were remained unchanged (*P* > 0.05) in the eggs of various located fish. Nonetheless, it is imperative to conduct additional research aimed at comprehending the precise factors responsible for driving these variations and assessing their potential implications for human health.

**Table 3:** Amino acid profile of Hilsa shad eggs sampled from three distinct regions of Bangladesh (*n* = 3).

Amino acids (%)	Locations		
	Cox’s Bazar	Chandpur	Patuakhali
Asp	8.60 ± 0.10^c^	9.07 ± 0.15^b^	9.50 ± 0.20^a^
Ser	9.10 ± 0.20^a^	6.73 ± 0.11^b^	6.27 ± 0.06^c^
Glu	14.60 ± 0.10^b^	14.93 ± 0.10^a^	14.20 ± 0.10^c^
Gly	3.87 ± 0.15^a^	3.47 ± 0.06^b^	3.40 ± 0.10^b^
Ala	12.77 ± 0.21	13.03 ± 0.12	13.00 ± 0.10
Cys	1.60 ± 0.10^c^	2.30 ± 0.10^b^	2.80 ± 0.10^a^
ΣNEAA	50.53 ± 0.21^a^	49.53 ± 0.21^b^	49.17 ± 0.21^b^
Thr	6.20 ± 0.10^a^	4.90 ± 0.10^b^	5.07 ± 0.15^b^
Val	4.93 ± 0.15^b^	5.37 ± 0.15^a^	5.00 ± 0.10^b^
Met	2.20 ± 0.10^b^	2.33 ± 0.06^b^	3.50 ± 0.10^a^
Ile	3.60 ± 0.10^b^	3.90 ± 0.10^a^	3.70 ± 0.10^b^
Leu	8.43 ± 0.21^b^	9.00 ± 0.10^a^	9.00 ± 0.10^a^
Tyr	3.47 ± 0.15^a^	3.60 ± 0.10^a^	3.20 ± 0.10^b^
Phe	3.50 ± 0.20	3.63 ± 0.25	3.33 ± 0.21
His	4.00 ± 0.10^b^	3.97 ± 0.15^b^	4.53 ± 0.25^a^
Lys	7.33 ± 0.11	7.60 ± 0.20	7.30 ± 0.10
Arg	5.80 ± 0.20	6.17 ± 0.25	6.20 ± 0.10
ΣEAA	49.47 ± 0.12^b^	50.47 ± 0.21^a^	50.83 ± 0.31^a^
EAA/NEAA	0.98 ± 0.01^b^	1.02 ± 0.01^a^	1.03 ± 0.02^a^

he data are represented as Mean ± SD of means.

Asp: Aspartic acid, Ser: serine, Glu: glutamic acid, Gly: glycine, Ala: alanine, Cys: cysteine, Thr: threonine, Val: valine, Met: methionine, Ile: isoleucine, Leu: leucine, Tyr: tyrosine, Phe: phenyl-alanine, His: histidine, Lys: lysine, Arg: arginine. Mean values with distinct superscripts specify remarkable variations (*P* < 0.05).

### Fatty acids composition in hilsa eggs roe

In this present investigation, a total of 22 fatty acids (including 10 SFAs, 5 MUFAs, and 7 PUFAs) were identified from the female Hilsa eggs roe located from Cox’s Bazar, Chandpur, and Patuakhali area (Table 4). Marine fish display species-specific variations in their fatty acid contents, affected by lot of factors related to fish size, age, nutrition, and environment^60^. Hilsa eggs from distinct locations revealed a higher dominance of ΣSFA (52.75–54.27%), followed by ΣMUFA (28.91–30.75%), and ΣPUFA (13.61–14.72%) (Table 4). However, Baki et al.^48^ found that Rainbow trout eggs from various locations contained ΣSFAs (27.16–31.77%), ΣMUFAs (25.32–29.14%), and ΣPUFAs (41.63–46.37%). Overall, the results indicated that various geographical locations significantly (*P* < 0.05) influenced the majority of the FAs compositions in fish eggs.

**Table 4:** Fatty acids composition (% fatty acids) in *T. ilisha* eggs sourced from three distinct locations of Bangladesh (*n* = 3).

Fatty acids	Locations		
	Cox’s Bazar	Chandpur	Patuakhali
C8:0 (caprylic acid)	0.31 ± 0.00^b^	0.31 ± 0.12^b^	0.53 ± 0.02^a^
C10:0 (capric acid)	0.02 ± 0.01^c^	0.31 ± 0.04^b^	0.39 ± 0.04^a^
C12:0 (lauric acid)	0.68 ± 0.07^a^	0.44 ± 0.08 ^b^	0.64 ± 0.06^a^
C13:0 (tridecylic acid)	3.51 ± 0.30^a^	2.64 ± 0.25^b^	3.98 ± 0.19^a^
C14:0 (myristic acid)	7.36 ± 0.13^b^	8.47 ± 0.25^a^	7.42 ± 0.05^b^
C16:0 (palmitic acid)	32.86 ± 0.39^a^	31.08 ± 0.24^b^	31.60 ± 1.02^b^
C18:0 (stearic acid)	9.10 ± 0.14^ab^	9.31 ± 0.35^a^	8.45 ± 0.50^b^
C20:0	0.09 ± 0.02	0.58 ± 0.52	0.02 ± 0.25
C21:0 (heneicosylic acid)	0.14 ± 0.13	0.11 ± 0.09	0.34 ± 0.20
C22:0 (behenic acid)	0.21 ± 0.04^ab^	0.55 ± 0.34^a^	0.07 ± 0.07^b^
ΣSFA	54.27 ± 0.06^a^	53.80 ± 1.11^ab^	52.75 ± 0.16^b^
C16:1 (palmitoleic acid)	7.49 ± 0.04^a^	7.13 ± 0.10^b^	7.43 ± 0.11^a^
C18:1 n-9 (oleic acid)	20.73 ± 0.40^b^	21.00 ± 0.74^b^	22.23 ± 0.57^a^
C17:1 (heptadecenoic acid)	0.35 ± 0.26	0.45 ± 0.09	0.65 ± 0.09
C19:1 (nonadecenoic acid)	0.27 ± 0.14	0.48 ± 0.13	0.42 ± 0.01
C24:1 (nervonic acid)	0.08 ± 0.04^ab^	0.09 ± 0.01^a^	0.04 ± 0.01^b^
ΣMUFA	28.91 ± 0.36^b^	29.15 ± 0.87^b^	30.75 ± 0.37^a^
C18:2n-6 (linoleic acid)	0.78 ± 0.15	0.96 ± 0.16	0.87 ± 0.06
C18:3n-6 (gamma-linolenic acid)	1.61 ± 0.38	1.43 ± 0.20	1.50 ± 0.08
C20:4n-6 (arachidonic acid)	0.35 ± 0.03	0.42 ± 0.40	0.17 ± 0.03
C20:5n-3, EPA (eicosapentaenoic acid)	5.12 ± 0.28	5.25 ± 0.75	5.99 ± 0.25
C20:3 n-6 (dihomo-gamma-linolenic acid)	0.10 ± 0.03^b^	0.72 ± 0.06^a^	0.50 ± 0.31^a^
C22:6 n-3, DHA (docosahexaenoic acid)	5.62 ± 0.84	5.55 ± 0.58	5.68 ± 0.06
C22:5 n-3 (docosapentaenoic acid)	0.05 ± 0.01	0.08 ± 0.06	0.02 ± 0.01
ΣPUFA	13.61 ± 0.05^c^	14.14 ± 0.28^b^	14.72 ± 0.07^a^
PUFA/SFA	0.25 ± 0.00	0.26 ± 0.00	0.28 ± 0.00
Σω-3 (total omega-3 fatty acids)	7.38 ± 3.10^b^	10.87 ± 0.11^ab^	11.68 ± 0.20^a^
Σω-6 (total omega-6 fatty acids	2.83 ± 0.52	3.27 ± 0.38	3.03 ± 0.27
n-3/n-6	2.69 ± 1.19	3.36 ± 0.43	3.87 ± 0.41
Others	3.21 ± 0.25^a^	2.91 ± 0.51^a^	1.79 ± 0.27^b^

The data are represented as mean ± sd of means.

Mean values with distinct superscripts specify remarkable variations (*P* < 0.05).

SFAs in fish eggs serve as an energy source and maintain cell membrane integrity. This study detected SFAs ranging from C8:0 to C22:0. The SFA profiles such as C8:0, C10:0, C12:0, C13:0, C14:0, C16:0, C18:0, and C22:0 had shown significant (*P* < 0.05) variations among the fish belongs to diverse locations (Table 4). These outcomes may be accredited to differences in environmental salinity, dietary lipid sources, and metabolic activity of broodstock, which affect fatty acid synthesis and deposition in the developing oocytes. Likewise, significant differences were also noted in T. *ilisha*^49^ *Oncorhynchus mykiss*^48^ and *Tenualosa* sp^50^. , when captured from different localities. Nevertheless, *T. ilisha* lacks C8:0, C10:0, C12:0, and C13:0^49^, certain Indian fish species lack C8:0, C10:0, and C22:0^61^, *T. ilisha* lacks C8:0, C10:0, and C12:0^3^, and *Oncorhynchus mykiss* lacks C8:0 and C10:0 ^48^. Previous research^3,35,48,62,63^ consistently showed a higher distribution of C16:0 in the eggs of several fishes, aligning with our finding. However, SFAs like C20:0 and C21:0 were followed no significant (*P* > 0.05) alterations in fish eggs in this study.

MUFAs offer several health benefits, especially in mitigating cardiovascular and inflammatory disorders^64^. The outcomes of this study specified that the mean values of MUFAs, particularly C16:1, C18:1 n-9 and C24:1 in Hilsa eggs roe were statistically (*P* < 0.05) differed among the various located fish (Table 4). These differences may be linked to regional variations in maternal diet, salinity-driven lipid metabolism, and energy requirements during gonadal maturation, which influence the synthesis and accumulation of specific MUFAs in the eggs. The present results were paralleled with Baki et al.^48^who found significant variations of these FAs composition in the eggs of Rainbow trout sourced from five distinct stations. Similarly, Mol and Turan^47^ noted significant differences in these MUFA levels in several kinds of eggs roe. In particular, C16:1 was significantly (*P* < 0.05) greater in Cox’s bazar (7.49%) and Patuakhali region (7.43%), while C18:1n-9 (22.73%) was noteworthy (*P* < 0.05) higher in Patuakhali area (Table 4), which approximated or even exceeded the levels found in the eggs of other fishes^3,44,48,65,66^. Meanwhile, among all MUFAs, C18:1n-9 (20.73–22.23%) was observed higher in Hilsa eggs than others, corroborated with the findings of Kowalska-Góralska et al.^44^ and Majdoubi et al.^65^. Conversely, the levels of C17:1 and C19:1 FAs in fish eggs were not affected by various geographical locations in this study. Baki et al.^48^ found remarkable changes of C17:1 level (0.36–0.58%) in *Oncorhynchus mykiss* eggs, although no studies have been observed to determine the C19:1 level in fish eggs.

PUFAs are essential for membrane structure, energy storage, immune function, proper development, and overall survival of the developing embryos^67^. In this study, the PUFAs contents in fish eggs such as C18:2n-6, C18:3n-6, C20:4n-6, C20:5n-3 EPA, C22:6n-3 DHA, and C22:5n-3 was numerically varied but did not show any significant (*P* > 0.05) variations among the fish from three distinct locations (Table 4), indicating almost identical PUFAs composition in fish eggs. However, Kowalska-Góralska et al.^44^ and Baki et al.^48^ documented substantial differences of these PUFAs levels in the eggs of Rainbow trout, and Salmonid as well as Acipenserid fish respectively. Dey et al.^68^ documented that n-3 HUFA, specifically EPA and DHA, are essential for growth, development, and survival of fish. Amongst all the PUFAs, C20:3n-6 was showed significant alternations in Hilsa eggs from various localities, which may be influenced by habitat-specific dietary inputs and physiological regulation of long-chain fatty acid biosynthesis. Similar variations were also investigated in others fish eggs, as noted by Hossain et al.^3^ and Kowalska-Góralska et al.^44^. In this study, significantly higher MUFA and PUFA in eggs of fish from Patuakhali region indicated that the eggs roe produced from this region is suitable for final product development, with increasing the taste, flavor, appearance, sweetness, and overall acceptability.

Based on the current findings, it was evident that the ratios of PUFA/SFA and n-6/n-3 were not significantly affected by various locations, and these values were lower than those reported in the muscle of *Tenualosa* sp^50^. The n-6/n-3 and PUFA/SFA ratios indicate nutritional status of fish, offering numerous health benefits for human^69^. The significantly (*P* < 0.05) highest Σω-3 (11.68 ± 0.20%) were detected in the eggs roe from Patuakhali area, whereas the Σω-6 content did not show notable variation among the Hilsa shad groups. Hossain et al.^3^ reported that the total Σω-3 content (11.66–12.51%) remained significantly unaffected, but there was significant variation in the Σω-6 level (2.20–2.50%) in *T. ilisha* eggs when collected from two different habitats.

In summary, the variations in fatty acid composition observed in Hilsa shad eggs from different locations can be credited to a combination of environmental factors, dietary differences, and upward migration. Furthermore, the study provides valuable insights into the potential for regional variations in fish eggs’ fatty acid profiles, which can be of interest to both researchers and consumers seeking specific nutritional attributes in fish products.

### Mineral composition of eggs roe

Table 5 denotes the mineral content in eggs of Hilsa collected from three different locations including Cox’s bazar, Chandpur, and Patuakhali of Bangladesh. Despite the mineral contents in muscle of various fishes are extensively documented in many existing literatures^3,11,43,50,70–72^ the mineral composition of fish eggs from diverse locations is not yet fully comphrehended. Minerals play an essential role in supporting fish growth, reproductive progress, bone strenthening, and energy metabolism^3,73^.

**Table 5:** Mineral composition (% dry matter) in hilsa Shad eggs sourced from cox’s bazar, chandpur, and Patuakhali (*n* = 3).

Minerals (mg/100 g)	Locations		
	Cox’s Bazar	Chandpur	Patuakhali
P	294.00 ± 1.00^a^	256.00 ± 0.50^c^	278.97 ± 0.15^b^
K	687.50 ± 1.00^b^	650.00 ± 0.50^c^	787.67 ± 0.96^a^
Ca	18.70 ± 0.30^b^	30.07 ± 0.55^a^	17.67 ± 0.31^c^
Mg	79.00 ± 0.20^b^	102.00 ± 0.40^a^	70.00 ± 0.70^c^
Zn	17.00 ± 0.30^b^	10.60 ± 0.60^c^	96.00 ± 0.20^a^
Cu	1.70 ± 0.20^c^	5.10 ± 0.10^a^	3.60 ± 0.30^b^
Fe	24.80 ± 0.80^a^	14.40 ± 0.40^c^	22.60 ± 0.60^b^
Mn	1.87 ± 0.03^a^	1.29 ± 0.01^c^	1.40 ± 0.05^b^
Cr	1.23 ± 0.06^b^	0.29 ± 0.01^c^	1.66 ± 0.04^a^

The data are represented as mean ± sd of means.

P-phosphorus, K-potassium, Ca-calcium, Mg-magnesium, Zn-zinc, Cu-copper, Fe-iron, Mn-manganese, Cr-chromium. Mean values with distinct superscripts specify remarkable variations (*P* < 0.05).

This study’s results provided valuable insights into the mineral composition of *T. ilisha* eggs and revealed significant (*P* < 0.05) variations in the mineral profile of Hilsa eggs across different sampling sites. These present findings might be due to the variations in water chemistry, maternal diet, and mineral availability in each habitat, influencing how minerals are deposited in developing eggs. Amongst the various minerals assessed in this current observation, K (650.00-787.67 mg/100 g) was the most abundant within Hilsa eggs, followed by P (256.00-294.00 mg/100 g) and Mg (70.00-102.00 mg/100 g) (Table 5). The prominence of these minerals in Hilsa eggs suggests that Hilsa eggs roe can be a significant dietary source of essential minerals for human health welfare. The mean concentrations of K and Mg in Hilsa shad eggs in this study exhibited significant variances (*P* < 0.05) among the sampled groups, surpassing the results of the study conducted by Hossain et al.^3^. In general, K has fundamental role in muscle contraction, energy metabolism, and transmission of nerve impulse^50^ whereas Mg triggers the activation of over 600 enzymes, stabilizes cellular function, facilitates cell repair, supports RNA & DNA synthesis, and boosts antioxidant activity^74^. Notably, noteworthy (*P* < 0.05) higher mean values of P (294.00 mg/100 g), Fe (24.80 mg/100 g), and Mn (1.87 mg/100 g) within eggs were detected in the Cox’s bazar district than the other sampled areas, indicating good source of these essential minerals. Lanes et al.^75^ documented markedly variations in the concentration of P and Fe between wild and captive Atlantic cod eggs. However, DePeters et al.^76^ reported no remarkable variations of these mineral contents between Sturgeon eggs from the wild and those raised in captivity. P is essential for skeletal development and DNA & RNA synthesis, Fe is vital for hemoglobin production and various physiological processes, and Mn is invloved in the functioning of numerous enzymes.

In addition, the fish group from the Chandpur district exhibited significantly (*P* < 0.05) higher contents of Ca (30.07 mg/100 g), Mg (102.00 mg/100 g), and Cu (5.10 mg/100 g) compared to other locations (Table 5). In this current research, the recorded concentrations of Ca, Mg, and Cu in *T. ilisha* eggs were comparatively higher than those found in other food sources, such as 8.5 mg/100 g of Ca in fruits^77^ 3.9 mg/100 g of Mg in aromatic and medicinal plant^72^ and 0.001 mg/100 g of Cu in fruits^77^. Similarly, *T. ilisha* fish eggs sourced from Arabian Gulf and Bay of Bengal revealed noteworthy differences of these vital mineral components, as reported by Hossain et al.^3^. Ca is important for muscle contraction and healthy bone, Mg acts as a co-factor in many enzymatic reactions, and Cu is essential for maintenance and development of central nervous system^78^.

The mean concentrations of K, Zn, and Cr were highly significant (*P* < 0.05) amongst the sampled fish, notably showing elevated levels in fish sampled from the Patuakhali area. The levels of the minerals in Hilsha shad eggs recorded in this study were much higher than those in *Tenualosa* sp., with values ranging from 30.0 to 91.4 mg/100 g for K, 10.4-20.3 mg/100 g for Zn, and 0.04-0.12 mg/100 g for Cr^50^. However, in this study, the Hilsa eggs from Chandpur district had significantly lowered P (256.00 mg/100 g), K (650 mg/100 g), Zn (10.60 mg/100 g), Fe (14.40 mg/100 g), Mn (1.29 mg/100 g), and Cr (0.29 mg/100 g) content (*P* < 0.05). Overall, these results suggest that Hilsa fish eggs from distinct locations may offer superior nutritional benefits in terms of minerals, potentially making them more desirable from a dietary perspective.

### Sensory evaluation of cooked hilsa eggs roe

**Fig. 2:**
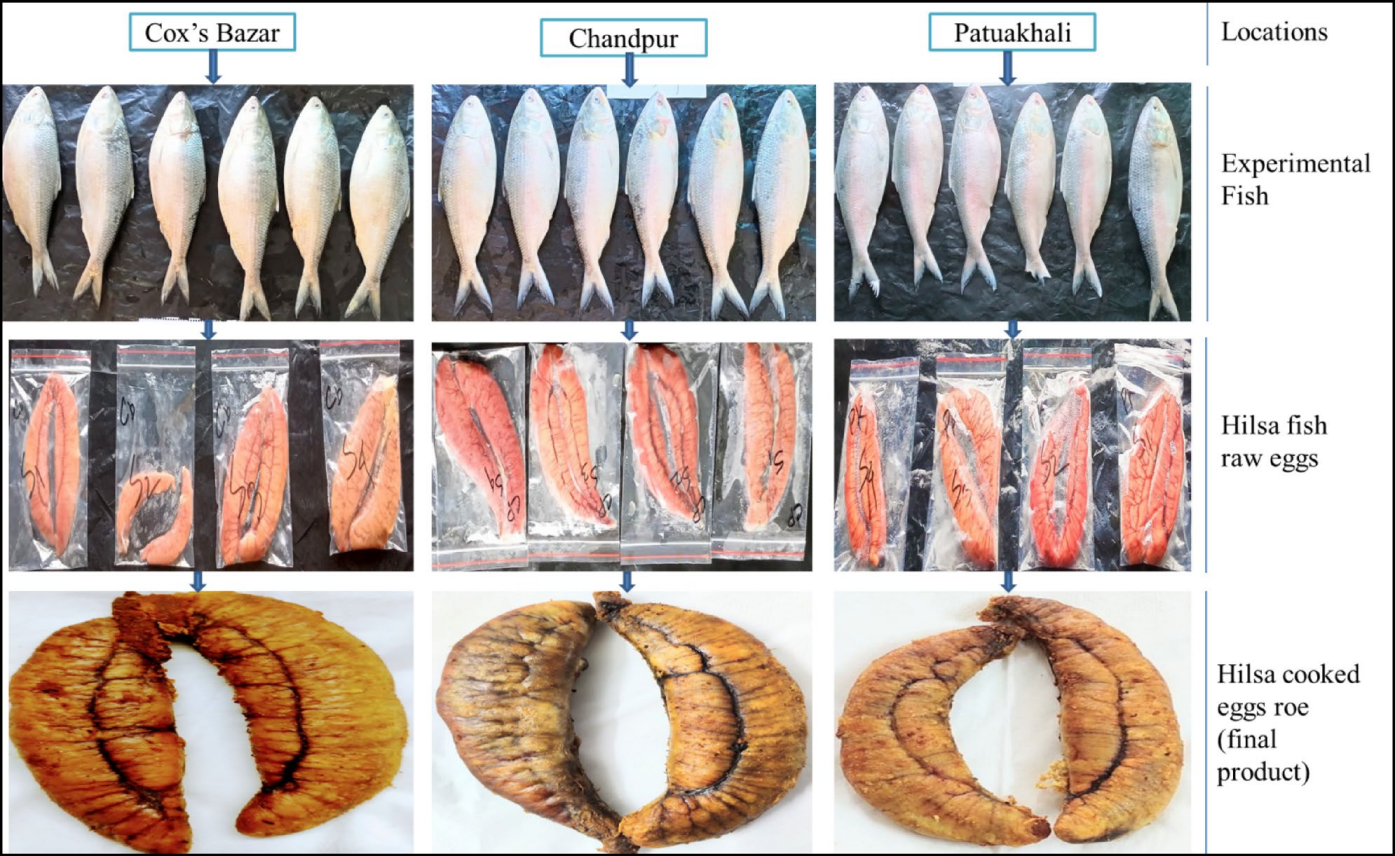
An overview of Hilsa eggs roe (final product) development process from diverse regions in Bangladesh.

Figure 2 provides the comprehensive illustration of the development process of *T. ilisha* eggs roe from 3 different locations of Bangladesh. This figure primarily emphasizes three distinct stages: (1) collection of experimental fish, (2) extraction of raw Hilsa eggs, and (3) subsequent development of Hilsa cooked eggs roe (final product).

**Fig. 3:**
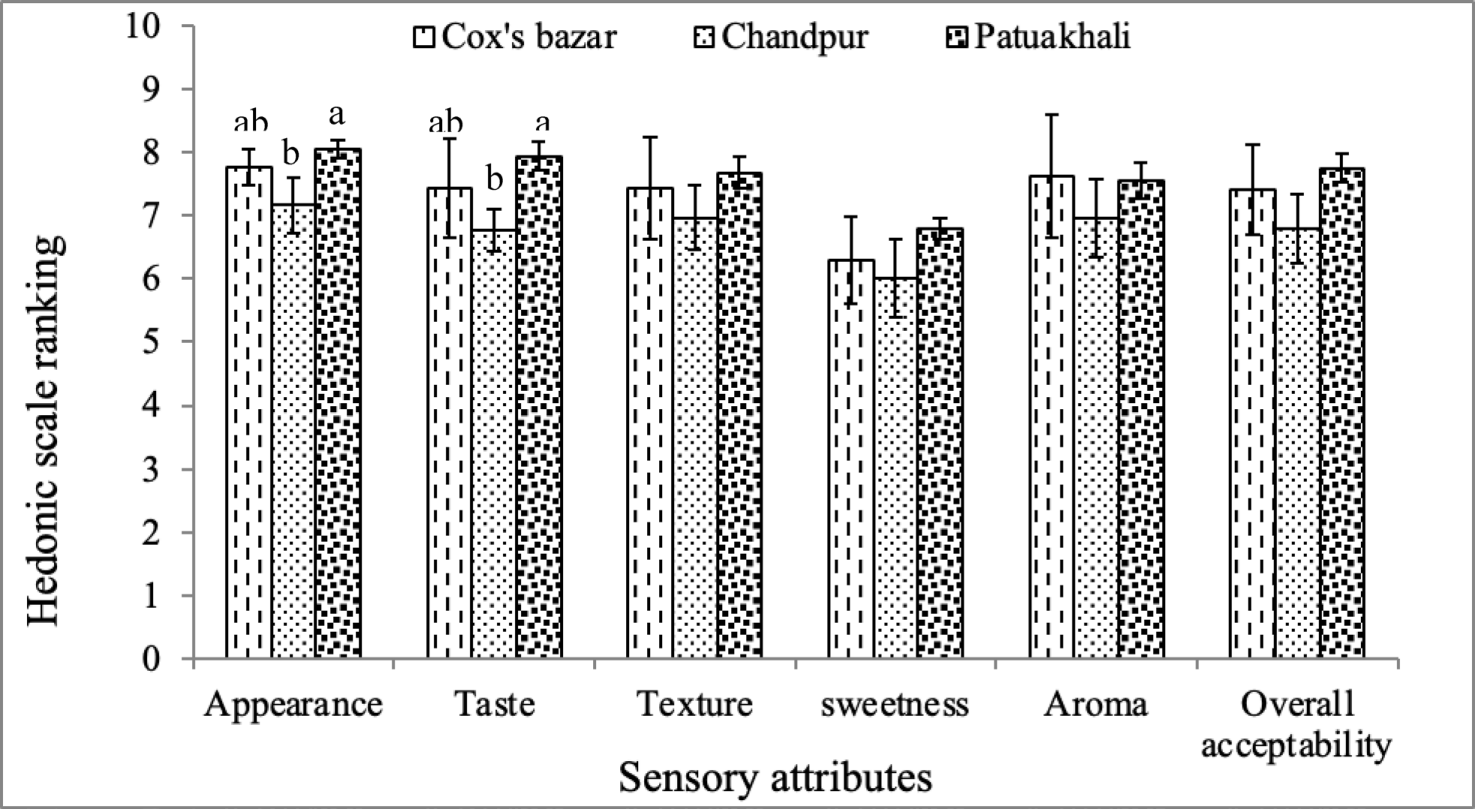
Sensory evaluation and consumer preference test of *T. ilisha* eggs roe product from three selected areas of Bangladesh (*n* = 30). The data are represented as Mean ± SD of means. Mean values with distinct superscripts specify remarkable variations (*P* < 0.05).

 The results, as depicted in Fig. 3, revealed that different geographical locations had significant (*P* < 0.05) impacts on certain sensory characteristics, particularly appearance and taste of Hilsa eggs roe in this study. Notably, the eggs roe from Patuakhali region exhibited significantly (*P* < 0.05) higher appearance (8.06 ± 0.14, *p*-value: 0.02) and taste (7.94 ± 0.23, *p*-value: 0.04), while no discernible changes were detected in these attributes in the eggs roe from Cox’s bazar. Conversely, significantly lower appearance and taste were noted in the eggs roe from Chandpur region. This implies that Patuakhali region might offer a favorable environment for producing eggs roe with enhanced sensory qualities in terms of appearance and taste. These sensory differences correspond closely with the nutritional composition data, indicating that higher MUFA and PUFA levels and favorable amino acid profiles in Patuakhali roe likely enhance its taste and appearance. De et al.^11^ found analogous outcomes in their research when investigating *T. ilisha* from the Hooghly and Padma rivers. Conversely, Chandpur region displayed significantly (*P* < 0.05) lower scores for these parameters across all locations, indicating that there may be challenges or suboptimal conditions affecting the sensory attributes of eggs roe in this area. The disparities noted in appearance and taste could likely be ascribed to a range of factors, including diverse environmental conditions, salinity, feeding patterns, and overall variations in the nutritional profile within these distinct regions.

 De et al.^11^ noted that Hilsa shad is highly regarded by consumers for its exceptional texture and flavor, making it a delectable fish options. Generally, a fish’s taste and flavor are highly affected by their feeding habit and dietary preference^79^. The unique taste of Hilsa fish is frequently accredited to its rich contents of fatty acids i.e., oleic acid, arachidonic acid, linolenic acid, linoleic acid, DHA, and EPA^10^. Furthermore, prior studies^11^ have noted that components such as glutamic acid, nucleotide, as well as sodium and chloride ions contributed to the flavor and taste of fish.

In addition, other sensory attributes including texture (6.97–7.68, *p*-value: 0.20), aroma (6.95–7.62, *p*-value: 0.24), sweetness (6.01–6.80, *p*-value: 0.21), and overall acceptability (6.80–7.75, *p*-value: 0.09) remained unaffected (*P* > 0.05) by geographical variations among Cox’s bazar, Chandpur, and Patuakhali. This suggests that these particular characteristics are less susceptible to the influence of geographical factors. Nonetheless, De et al.^11^ revealed notable variations in the texture and overall acceptability of Hilsa fish. However, the eggs roe from Patuakhali region achieved numerical higher ratings for these attributes in comparison with other sampled locations. It’s worth highlighting that the upstream migratory fishes including Hilsa shad, Chum salmon, Atlantic salmon, and Sockeye salmon primarily transform SFA into MUFA and PUFA during migration^80^. High levels of MUFA and PUFA, particularly oleic acid, ω-3, and ω-6 FAs, significantly contribute to the excellent texture & aroma in fish^11^. The numerical superior texture and aroma scores in eggs from Patuakhali region might be due to the elevated concentrations of these fatty acids. Correspondingly, in this study, the eggs roe developed from the Patuakhali area also showed a numerically greater sweetness, possibly attributed to the abundance of certain amino acids, as reported by Fuke and Konosu^81^. The panel response consistency was confirmed by analyzing standard deviation of means (SD), which remained within acceptable ranges for all attributes, indicating reliable scoring across panelists. Overall, the stability in majority of the sensory parameters among different regions may also have implications for product standardization and consumer preferences. In summary, Hilsa eggs roe from Patuakhali exhibited the best combined nutritional and sensory qualities, supporting its potential as a superior product for commercialization and consumer acceptance.

## Conclusion

 Based on the findings of this study, it can be concluded that Hilsa eggs roe from three different geographical regions represents a rich source of essential dietary nutrients such as protein, lipid, amino acids, fatty acids, and minerals composition, enriching the nutrient composition knowledge base data bank. In particular, Hilsa egg roe development from Patuakhali region showed significantly higher amounts of certain valuable nutrients such as amino acids (Asp, Cys, Met, Leu, His, ΣEAA, EAA/NEAA), fatty acids (C8:0, C10:0, C12:0, C13:0, C16:1, C18:1 n-9, C20:3 n-6, and Σω-3) and minerals (K, Zn, and Cr), as well as sensory attributes (appearance and taste). These findings may guide nutritional recommendations and create consumer awareness regarding the dietary benefits of Hilsa roe. However, considering the species’ ecological and reproductive importance, the harvesting of roe should be carefully regulated. Uncontrolled collection could adversely affect Hilsa breeding populations and long-term population stability. Thus, any future utilization of Hilsa roe should follow sustainable harvesting practices to ensure species conservation. With appropriate safeguards, Hilsa roe holds promise as a novel, nutritious product that could contribute to economic opportunities in various regions of Bangladesh and support value-added diversification in the fisheries sector, without compromising biodiversity.

 In addition, this study makes a significant scientific contribution by providing the first comprehensive comparative analysis of the nutritional and sensory profiles of Tenualosa ilisha eggs roe sourced from distinct geographic regions of Bangladesh. The findings offer valuable insights that can directly inform fishery product development, enabling the selection of superior quality roe for commercialization based on region-specific nutritional advantages. Moreover, the detailed nutrient composition data can aid dieticians in making targeted dietary recommendations and support aquaculture nutritionists in formulating optimized broodstock diets to enhance egg quality. For future research, it is recommended to explore shelf-life stability of Hilsa roe, conduct product development trials to diversify value-added products, and undertake broader consumer preference assessments to better align product attributes with market demand.

## Data Availability

The datasets used and/or analysed during the current study are available from the corresponding author upon reasonable request.
